# The association between dynamic changes in vitamin D and frailty alterations: A prospective analysis of UK Biobank participants

**DOI:** 10.1002/jcsm.13525

**Published:** 2024-06-24

**Authors:** Pan Zhang, Jinghui Zhong, Xinfeng Liu, Wen Sun

**Affiliations:** ^1^ Department of Neurology, Centre for Leading Medicine and Advanced Technologies of IHM, The First Affiliated Hospital of USTC, Division of Life Sciences and Medicine University of Science and Technology of China, Hefei, China Hefei China

**Keywords:** Dynamic changes in vitamin D, Frailty, Frailty risk factors, Transition, Vitamin D

## Abstract

**Background:**

Frailty is a common geriatric syndrome associated with reduced reserves and increased vulnerability to stressors among older adults. Vitamin D deficiency has been implicated in frailty, as it is essential for maintaining musculoskeletal functions. The relationship between dynamic changes in vitamin D levels and frailty over time has not been extensively studied.

**Methods:**

This study utilized data from the UK Biobank. Baseline and longitudinal changes in vitamin D levels were measured. Frailty status was assessed using both the frailty phenotype and frailty index approaches and classified as robust, pre‐frail, or frail. Changes in frailty status were assessed by frailty phenotype at baseline (2006–2010) and the follow‐up (2012–2013). Mixed effect model was performed to examine the association between vitamin D levels and frailty status. Using multistate transition models, we assessed the impact of increasing vitamin D levels on the probabilities of transitioning between robust, pre‐frail, and frail states.

**Results:**

Based on the frailty phenotype, 287 926 individuals (64.8%) were identified as having various degrees of frailty (median age 58.00 [51.00, 64.00] years, 55.9% females). Using the frailty index approach, 250 566 individuals (56%) were found to have different levels of frailty (median age 59.00 [51.00, 64.00] years, 55.3% females). Baseline vitamin D levels were found to be significantly associated with frailty status (frailty phenotype: OR_frail_ 0.78, 95% CI [0.76, 0.79], *P* < 0.001; frailty index: OR_frail_ 0.80, 95% CI [0.78, 0.81], *P* < 0.001). Dynamic changes in vitamin D levels were also found to be associated with changes in frailty over time. Furthermore, increasing vitamin D levels were associated with a transition from frailty to a healthier status. A higher degree of vitamin D (estimated at 1 nmol/L) was associated with a lower risk of transitioning from robust to prefrail (HR 0.997, 95% CI [0.995, 0.999]) and from prefrail to frail (HR 0.992, 95% CI [0.988, 0.995]).

**Conclusions:**

This study highlights the importance of vitamin D in the context of frailty. Low vitamin D levels are associated with increased frailty risk, while increasing vitamin D levels may contribute to improving frailty status. Recognizing the relationship between vitamin D levels and frailty can inform personalized management and early interventions for frail individuals. Further research is needed to explore the potential effects of vitamin D interventions on frailty and deepen our understanding of the biological connections between vitamin D and frailty.

## Introduction

As the global population ages, the prevalence of frailty, a common geriatric syndrome, is increasing and becoming a significant health burden.^1^, ^2^ Frailty is characterized by reduced reserves across multiple physiological systems, leading to increased vulnerability to stressors.^2^, ^3^ Several operational definitions of frailty exist, with two of the most widely used being the frailty index and the frailty phenotype. The frailty index involves counting age‐related health deficits, while the frailty phenotype is a specific syndrome determined by a combination of low grip strength, weight loss, exhaustion, low physical activity, and slow walking pace.^4^, ^5^ Both measures aim to identify vulnerability to physiological decompensation, setting them apart from related concepts such as multimorbidity.^6^

Frailty has been shown to significantly increase the risk of adverse health outcomes, including disability, falls, morbidity, long‐term care utilization, and mortality.^7^ Additionally, research has demonstrated a strong association between frailty and other conditions such as depression, cardiovascular disease (CVD), and dementia.^8^, ^9^, ^10^ Therefore, recognizing the potential relationship between frailty and factors that can modify frailty status would be advantageous for frail individuals, as it would enable them to pursue personalized management and early interventions.

Vitamin D is essential for maintaining musculoskeletal functions.^11^ In older adults, vitamin D deficiency can lead to reduced muscle strength and low bone mineral density.^11^ Frail individuals often have inadequate vitamin D levels. Previous studies have shown that vitamin D deficiency increases the risk of frailty and pre‐frailty.^12^ However, these studies only considered baseline measurements and did not examine the relationship between changes in vitamin D levels and frailty over time. Importantly, emerging evidence suggests that frailty may be reversible through appropriate interventions.^13^ Therefore, it is crucial to investigate the potential links between changes in vitamin D levels and the risk of developing frailty.

The aim of this study was to investigate the combined impact of baseline and longitudinal changes in vitamin D levels on incident frailty status. Using data from the UK Biobank, we employed two different definitions of frailty to investigate the relationship between baseline vitamin D levels and frailty, as well as to examine the association between dynamic changes in Vitamin D and changes in frailty. Furthermore, we aimed to assess how increasing levels of vitamin D are associated with transitioning between different frailty status.

## Method

### Study population

This study was a prospective, population‐based cohort study conducted using data from participants enrolled in the UK Biobank. A total of 502 640 participants were recruited through postal invitation between 2006 and 2010.^14^ The participants attended one of 22 assessment centres across England, Scotland, and Wales, where they completed a touchscreen questionnaire, underwent a nurse‐led interview, and had physical measurements taken. Written informed consent was obtained from all participants for data collection, analysis, and linkage. To be eligible for participation, individuals had to be registered with a general practitioner, reside within a reasonable travelling distance from the assessment center, and be between 40 and 69 years of age. In this study, wave 1 (2006–2010) was regarded as the baseline. Wave 2 (2012–2013) was regarded as the second survey. This particular study was conducted under the UKB Resource application number 106487.

### Sample collection and vitamin D concentration

During the recruitment and follow‐up process, blood samples were collected from each participant after an overnight fasting period and stored at −80°C prior to analysis.

Participants were categorized into four groups based on their serum vitamin D levels: <25 nmol/L, 25–<50 nmol/L, 50–<75 nmol/L, and ≥75 nmol/L.^15^

### Frailty definition

In this study, we employed two different approaches to assess frailty: the frailty phenotype and the frailty index.^5^, ^16^ Both assessments were performed during the baseline period (2006–2010). The frailty phenotype was also measured during the follow‐up period (2012–2013). The traits that make up the frailty index primarily consist of health deficits, many of which are long‐term morbidities and not easily modifiable. Using the frailty index may prevent us from establishing the dynamic changes in frailty. Therefore, in this study, we focused on observing the dynamic changes in frailty using the frailty phenotype assessment.

### Frailty phenotype

The Fried frailty phenotype evaluates five indicators to assess frailty: weight loss, exhaustion, low grip strength, physical inactivity, and slow walking speed (Table S1).^5^ The severity of frailty is determined by the number of criteria met, resulting in a score ranging from 0 to 5.

In our study, participants were categorized into three groups based on their fulfilment of the criteria: frail, pre‐frail, and robust. Frail individuals were classified as those who met three or more of the five criteria, pre‐frail individuals met one or two criteria, and robust individuals did not meet any of the criteria. These three groups were mutually exclusive.

### Frailty index

The frailty index approach, developed by Rockwood and Mitnitski, is a comprehensive assessment of deficits that includes routine clinical data such as symptoms, signs, disabilities, and diseases that meet standardized criteria.^4^, ^17^ It calculates the proportion of total health deficits a person has by dividing the total number of deficits they exhibit by the total number of measurable deficits, resulting in a score between 0 and 1. Higher scores indicate a higher degree of frailty.

In our study, we employed a previously validated frailty index comprising 49 self‐reported questionnaire items related to health, disease presence, disability, and mental well‐being (Table S2).^16^ Using this frailty index, participants were classified into three categories based on their frailty status: robust (FI ≤ 0.10), pre‐frail (0.10 < FI < 0.25), and frail (FI ≥ 0.25).

Detailed definitions of the frailty phenotype and the frailty index can be found in Tables S1 and S2.

### Covariates

Participants' age, sex, smoking status, and drinking status were obtained using a self‐completed touchscreen questionnaire. Smoking status and drinking status were categorized into three groups: never, previous, and current. To assess socioeconomic deprivation, the Townsend deprivation index was calculated based on the participant's postcode and the preceding national census output areas.^18^ A higher score on this index indicates a higher level of socioeconomic deprivation. Ethnicity was self‐reported and categorized into white, black, Asian, and other or mixed ethnic background. Participants also self‐reported their hours of sleep, which were categorized as normal (7–9 h), long (>9 h), or short (<7 h).

In addition, we collected information on sunshine exposure time, vitamin D supplementation status, and weekly exercise time.

### Statistical analysis

Descriptive characteristics are reported using means and standard deviations (SDs) for quantitative variables that followed a normal distribution. For variables that were not normally distributed, medians and interquartile ranges (IQRs) were used. Categorical variables are presented as frequencies and percentages.

To investigate the association between baseline vitamin D levels and the risks of frailty status and individual frailty phenotype items, a mixed effects model was employed with the assessment centre considered as a random effect. The odds ratio (OR) and its corresponding 95% confidence interval (CI) were calculated. Three models were fitted, with robust participants serving as the reference group. Model 1 adjusted for age, sex, and ethnicity. Model 2 included additional multivariable adjustments for smoking status, drinking status, and the Townsend deprivation index. Model 3 further adjusted for physical activity time, vitamin D supplementation, and sunshine exposure time. Similarly, we used the same methodology to analyse the associations between changes in vitamin D levels and changes in frailty status. Vitamin D levels at baseline and follow‐up were categorized into four groups: stable low, stable high, increase, and decrease, based on a cutoff of 75 nmol/L. The changes in frailty status can be categorized as stable robust, robust to prefrail/frail, prefrail to robust, prefrail to frail, frail to robust/prefrail, and stable prefrail/frail.

To examine the non‐linear relationships between vitamin D levels and frailty risk, we employed restricted cubic spline analysis. Furthermore, we utilized multistate Markov models to analyse the associations between baseline vitamin D levels and the probabilities of transitioning between different frailty states. The hazard ratio (HR) and its corresponding 95% CI were calculated. In addition, we generated predictive probability plots to visualize the relationship between vitamin D levels and frailty status.

We also conducted subgroup analyses to assess the impact of vitamin D on frailty status in sex (men vs. women) and age (<60 vs. ≥60). In addition, we created probability graphs to examine the occurrence of prefrailty and frailty in different sexes and age groups as vitamin D levels changed.

Several sensitivity analyses were conducted: (i) To account for sample attrition, an inverse probability of attrition weighting (IPAW) analysis was employed. This analysis estimated the probability of frailty status at baseline and follow‐up using multivariate‐adjusted logistic regressions. The inverse of these probabilities was then utilized as weights in the corresponding analysis, ensuring that attrition did not bias the results. (ii) To handle missing values for covariates, individuals with missing data were excluded from the analysis, and the analysis was conducted using the complete dataset.

All statistical analyses were conducted using R software (Version 4.1.2). *P*‐values were two‐sided, and *P* < 0.05 was considered statistically significant.

## Results

### Baseline characteristics of the study population

**Figure 1:**
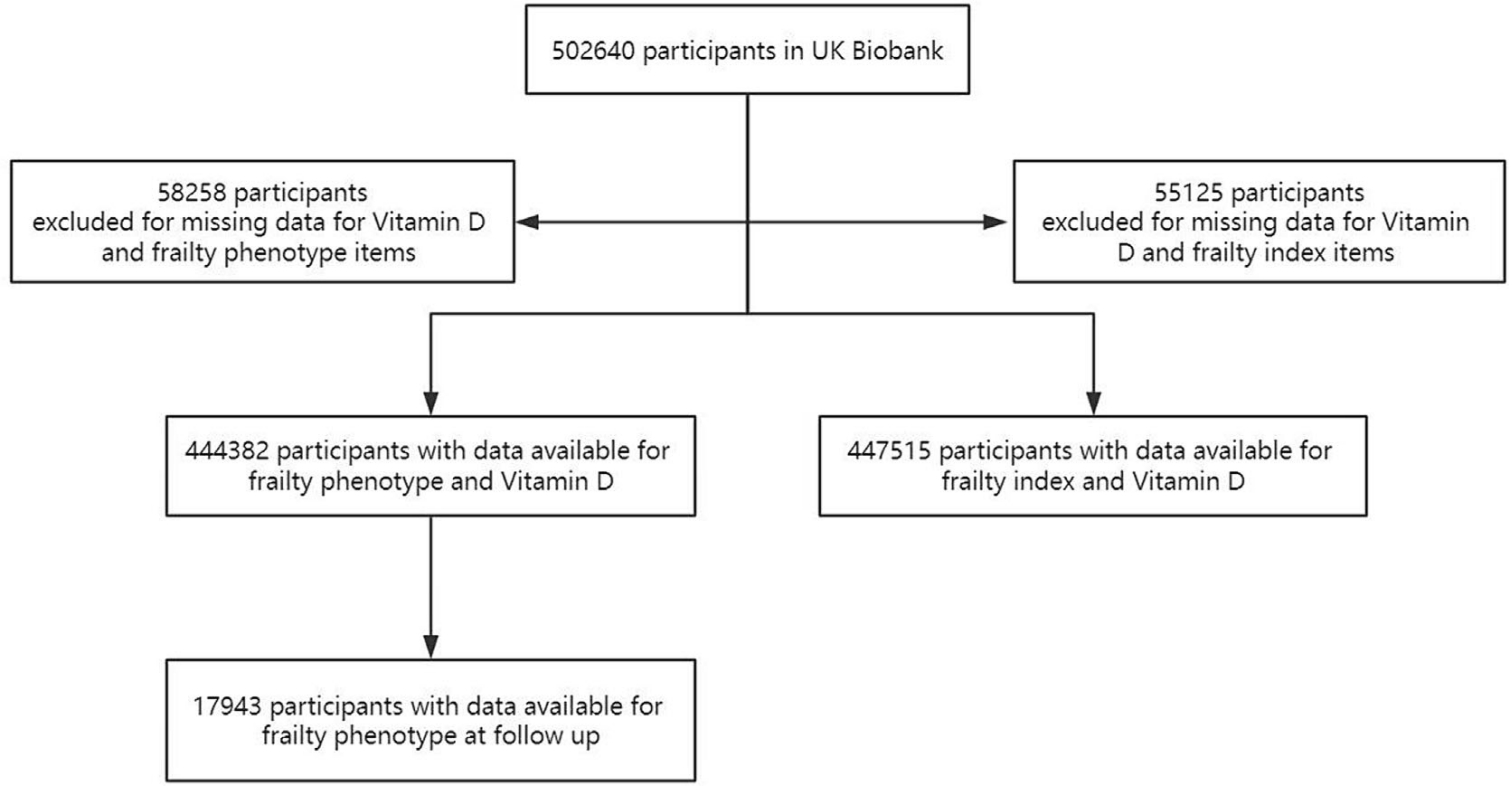
Flow chart.

After excluding participants with missing data for vitamin D and frailty phenotype items, a total of 444 382 participants were included in the current study for the analysis of frailty phenotype (Figure 1). Among them, 35 697 (8%) participants met the criteria for frailty, while 252 229 (56.8%) participants were categorized as pre‐frail.

For the analysis of frailty index, after excluding participants with missing data for vitamin D and frailty index items, a total of 447 515 participants were included (Figure 1). Among them, 21 122 (4.7%) participants met the criteria for frailty, while 229 444 (51.3%) participants were categorized as pre‐frail.

The baseline characteristics of the frailty phenotype are presented in Table 1, and the baseline characteristics of the frailty index categories are shown in Table S3. Both definitions of frailty showed that frail individuals were older, more likely to be women, and had lower levels of vitamin D (*P* < 0.001).

**Table 1:** Baseline characteristics by frailty category at baseline and follow up

Characteristic	Frailty phenotype at baseline				Frailty phenotype at follow up			
	Robust	Pre‐frail	Frail	*P*	Robust	Pre‐frail	Frail	*P*
No. of participants	15 6456 (35.2)	25 2229 (56.8)	35 697 (8.0)		4924 (27.4)	11 475 (64)	1544 (8.6)	
Age at baseline, years	56.00 [49.00, 62.00]	58.00 [50.00, 63.00]	59.00 [53.00, 64.00]	<0.001	57.00 [50.00, 62.00]	59.00 [53.00, 63.00]	59.00 [54.00, 64.00]	<0.001
Sex				<0.001				<0.001
Female	77 129 (49.3)	138 814 (55.0)	22 221 (62.2)		2076 (42.2)	6003 (52.3)	932 (60.4)	
Male	79 327 (50.7)	113 415 (45.0)	13 476 (37.8)		2848 (57.8)	5472 (47.7)	612 (39.6)	
Townsend deprivation index, median (IQR)	−2.43 [−3.80, −0.14]	−2.11 [−3.63, 0.56]	−0.72 [−2.95, 2.58]	<0.001	−2.94 [−4.11, −1.11]	−2.72 [−3.96, −0.80]	−2.18 [−3.70, 0.29]	<0.001
Smoking status				<0.001				0.001
Never	88 097 (56.4)	136 966 (54.5)	17 486 (49.4)		2977 (60.5)	6725 (58.7)	841 (54.7)	
Former	54 024 (34.6)	87 620 (34.9)	12 317 (34.8)		1649 (33.5)	4019 (35.1)	577 (37.5)	
Current	14 030 (9.0)	26 507 (10.6)	5603 (15.8)		292 (5.9)	704 (6.1)	120 (7.8)	
Alcohol status				<0.001				<0.001
Never	4195 (2.7)	11 347 (4.5)	3340 (9.4)		107 (2.2)	346 (3.0)	81 (5.2)	
Former	3746 (2.4)	9006 (3.6)	2988 (8.4)		94 (1.9)	272 (2.4)	84 (5.4)	
Current	148 463 (94.9)	231 474 (91.9)	29 218 (82.2)		4722 (95.9)	10 854 (94.6)	1378 (89.3)	
Ethnicity				<0.001				<0.001
White	151 795 (97.3)	239 923 (95.5)	32 407 (91.3)		4846 (98.5)	11 233 (98.1)	1491 (96.8)	
Black	664 (0.4)	1843 (0.7)	485 (1.4)		9 (0.2)	21 (0.2)	8 (0.5)	
Asian	1845 (1.2)	5626 (2.2)	1710 (4.8)		36 (0.7)	95 (0.8)	17 (1.1)	
Mixed	897 (0.6)	1465 (0.6)	249 (0.7)		13 (0.3)	38 (0.3)	15 (1.0)	
Other	854 (0.5)	2382 (0.9)	638 (1.8)		15 (0.3)	58 (0.5)	9 (0.6)	
Sleep time				<0.001				<0.001
Short	32 575 (20.8)	63 902 (25.5)	12 228 (35.1)		937 (19.0)	2490 (21.8)	433 (28.4)	
Normal	122 405 (78.3)	182 271 (72.8)	20 489 (58.8)		3952 (80.3)	8813 (77.1)	1038 (68.2)	
Long	1314 (0.8)	4327 (1.7)	2120 (6.1)		30 (0.6)	130 (1.1)	52 (3.4)	
Baseline vitamin D	49.80 [35.10, 64.80]	46.10 [31.90, 61.70]	39.10 [26.00, 55.80]	<0.001	51.30 [35.98, 65.80]	47.60 [33.70, 62.00]	41.70 [28.67, 56.50]	<0.001

### Association of baseline vitamin D with frailty status

When using the frailty phenotype definition, compared to robust individuals, higher baseline vitamin D levels were associated with a lower risk of being pre‐frail (OR 0.82, 95% CI 0.82, 0.83) and frail (OR 0.58, 95% CI 0.57, 0.59) in Model 1. These associations were slightly attenuated but still significant after adjusting for vitamin D supplementation and other factors in Model 3 (OR pre‐frail 0.90, 95% CI 0.90, 0.91; OR frail 0.78, 95% CI 0.76, 0.79) (Table 2). Similar results were observed when using the frailty index (Table 2).

**Table 2:** Association between baseline vitamin D and frailty status

	Robust	Pre‐frail	*P*	Frail	*P*
Frailty phenotype					
Model 1, aOR (95% CI)	1 [Reference]	0.82 [0.82, 0.83]	<0.001	0.58 [0.57, 0.59]	<0.001
Model 2, aOR (95% CI)	1 [Reference]	0.85 [0.84, 0.85]	<0.001	0.65 [0.64, 0.66]	<0.001
Model 3, aOR (95% CI)	1 [Reference]	0.90 [0.90, 0.91]	<0.001	0.78 [0.76, 0.79]	<0.001
Frailty index					
Model 1, aOR (95% CI)	1 [Reference]	0.90 [0.90, 0.91]	<0.001	0.68 [0.67, 0.69]	<0.001
Model 2, aOR (95% CI)	1 [Reference]	0.93 [0.92, 0.94]	<0.001	0.77 [0.76, 0.78]	<0.001
Model 3, aOR (95% CI)	1 [Reference]	0.94 [0.93, 0.94]	<0.001	0.80 [0.78, 0.81]	<0.001

Mode 1 adjusted age, sex, and ethnicity. Mode 2 adjusted age, sex, ethnicity, smoking status, drinking status, and the Townsend deprivation index. Mode 3 adjusted age, sex, ethnicity, smoking status, drinking status, the Townsend deprivation index, physical activity time, vitamin D supplementation, and sunshine exposure time.

Table S4 presents the demographic characteristics of individuals categorized by serum vitamin D levels. Among them, 394 335 individuals (88.7%) were categorized as having insufficient vitamin D levels, while 59 326 individuals (13.3%) were classified as deficient in vitamin D.

Compared with the group with sufficient serum vitamin D levels (≥75 nmol/L), individuals with lower serum vitamin D levels demonstrated higher odds of being pre‐frail. Specifically, the ORs and corresponding 95% CIs were as follows: 50–75 nmol/L versus ≥75 nmol/L (OR 1.05, 95% CI 1.02, 1.07), 25–50 nmol/L versus ≥75 nmol/L (OR 1.18, 95% CI 1.16, 1.21), and <25 nmol/L versus ≥75 nmol/L (OR 1.41, 95% CI 1.36, 1.45). Similarly, individuals with lower serum vitamin D levels had higher odds of being frail compared with robust individuals: 50–75 nmol/L versus ≥75 nmol/L (OR 1.05, 95% CI 1.04, 1.36), 25–49 nmol/L versus ≥75 nmol/L (OR 1.38, 95% CI 1.29, 1.47), and <25 nmol/L versus ≥75 nmol/L (OR 2.15, 95% CI 2.00, 2.31) (Table S5). Similar results were observed in the analysis using the frailty index (Table S5).

**Figure 2:**
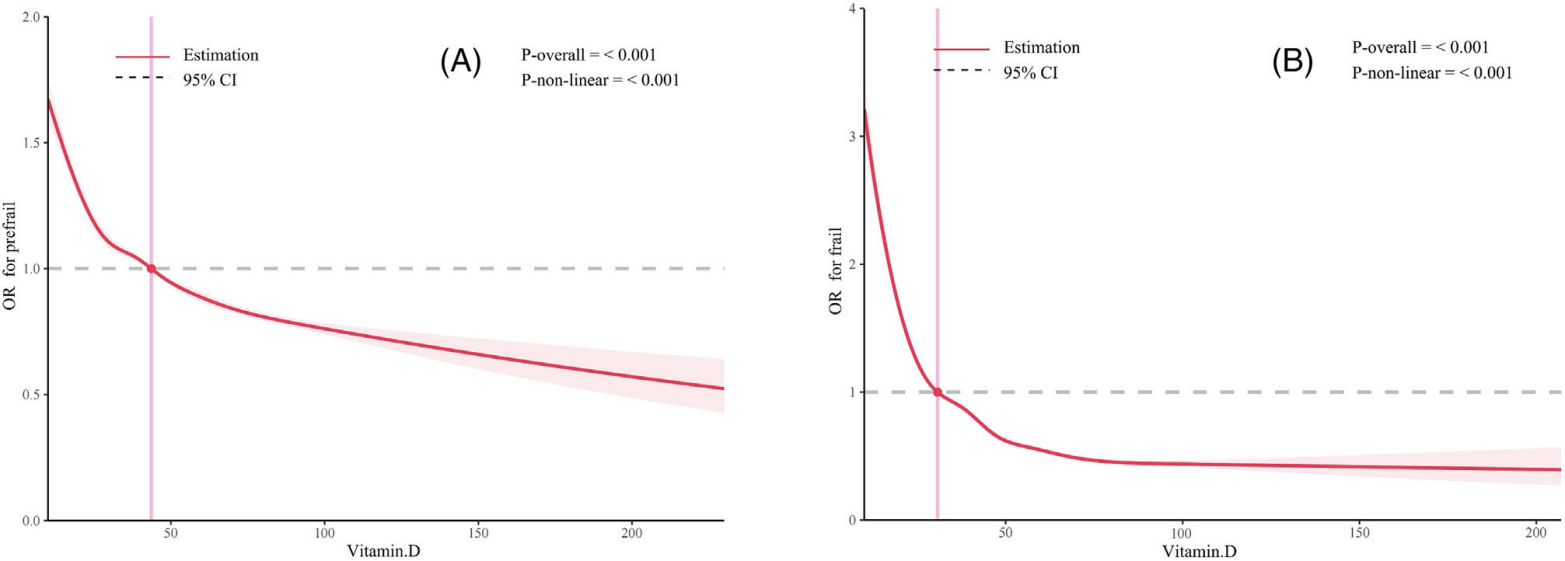
Restricted cubic spline of the association between vitamin D and frailty status. *(A)* For prefrail: The cutoff point at 43.618 nmol/L. *(B)* For frail: The cutoff point at 30.729 nmol/L.

Figure 2A illustrates the nonlinear ‘L‐shaped’ relationship between vitamin D concentration and the risk of pre‐frailty, with a gradual decrease in risk. A cutoff point at 43.618 nmol/L was identified, indicating a higher risk of pre‐frailty below this threshold. Similarly, Figure 2B shows a nonlinear ‘L‐shaped’ pattern between vitamin D concentration and the risk of frailty, with a cutoff point at 30.729 nmol/L, below which the risk of frailty is higher.

Figure S1 displays the probability of frailty status (stable, pre‐frail, and frail) in relation to vitamin D levels. As vitamin D concentration increases, the probability of being in the stable category increases, while the probabilities of being pre‐frail and frail decrease.

### Association of baseline vitamin D with components of frailty phenotype

Elevated serum levels of vitamin D were associated with an increased risk of weight loss (OR 1.06, 95% CI 1.05, 1.07), a decreased risk of exhaustion (OR 0.85, 95% CI 0.84, 0.86), a decreased risk of low grip strength (OR 0.90, 95% CI 0.89, 0.90), a decreased risk of low physical activity (OR 0.83, 95% CI 0.83, 0.84), and a decreased risk of slow gait speed (OR 0.80, 95% CI 0.79, 0.81) (Table 3).

**Table 3:** Association between baseline vitamin D and individual components of frailty

Frailty component	Model 1, aOR (95% CI)	Model 2, aOR (95% CI)	Model 3, aOR (95% CI)
Weight loss	1.05 [1.04, 1.06]	1.06 [1.06, 1.07]	1.06 [1.05, 1.07]
Exhaustion	0.79 [0.78, 0.80]	0.83 [0.83, 0.84]	0.85 [0.84, 0.86]
Low physical activity	0.80 [0.80, 0.81]	0.81 [0.81, 0.82]	0.83 [0.83, 0.84]
Slow gait speed	0.68 [0.67, 0.69]	0.75 [0.74, 0.76]	0.80 [0.79, 0.81]
Low grip strength	0.85 [0.84, 0.85]	0.88 [0.87, 0.88]	0.90 [0.89, 0.90]

Mode 1 adjusted age, sex, and ethnicity. Mode 2 adjusted age, sex, ethnicity, smoking status, drinking status, and the Townsend deprivation index. Mode 3 adjusted age, sex, ethnicity, smoking status, drinking status, the Townsend deprivation index, physical activity time, vitamin D supplementation, and sunshine exposure time.

When further categorizing vitamin D as a variable, these associations remained consistent (Table S6).

### Association of dynamic changes in vitamin D with changes in frailty status

A total of 17 943 individuals had follow‐up data on frailty status, with 1544 (8.6%) classified as frail and 11 475 (64%) classified as prefrail. Baseline information regarding frailty status at follow is presented in Table 1.

**Figure 3:**
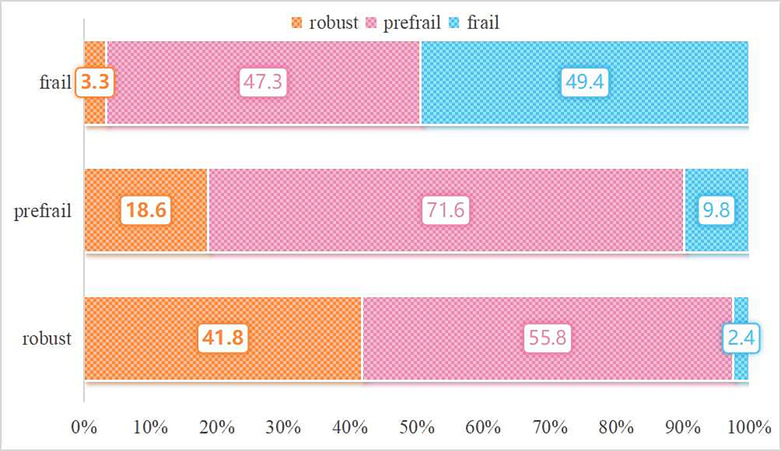
Distribution of frailty status transitions. The text on the left indicates the frailty state at baseline.

Figure 3 displays the quantitative and proportional changes in frailty status. Among the initial group of robust participants, 58.2% transitioning to pre‐frail or frail status. Conversely, among the initial group of frail participants, 50.6% exhibited an improvement, transitioning to pre‐frail or robust status. The baseline characteristics of the participants, categorized based on their change in frailty status, can be found in Table S7. The characteristics of the populations in serum vitamin D levels increased, remained the same, or decreased was presented in Table S8.

**Figure 4:**
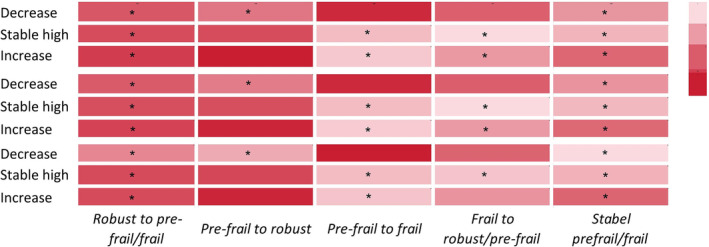
The association between change in vitamin D and change in frailty status. The depth of colour represents the magnitude of the odds ratio (OR), with darker shades indicating larger OR values. The symbol ‘*’ denotes statistical significance at *P* < 0. 05. The *y*‐axis represents the change in vitamin D levels. The *x*‐axis represents the change in frailty status. The first three lines represent the results of Model 1. The middle three lines represent the results of Model 2. The bottom three lines represent the results of Model 3.

Compared to stable low vitamin D, stable high vitamin D (OR 0.80, 95%CI[0.65, 0.99]) and decrease in vitamin D (OR 0.77, 95%CI[0.63, 0.93]) are less likely to transition from robust to pre‐frail/frail. Compared to stable low vitamin D, stable high vitamin D is also less likely to transition from pre‐frail to frail (OR 0.50, 95%CI[0.33, 0.77]). Stable high vitamin D is less likely to transition from frail to robust/pre‐frail (OR, 0.48, 95%CI[0.26, 0.90]). Vitamin D decrease is less likely to transition from pre‐frail to robust (OR 0.72, 95%CI[0.55, 0.93]). Vitamin D increase is less likely to transition from pre‐frail to frail (OR 0.48, 95%CI[0.32, 0.73]). Compared to stable low vitamin D, vitamin D decrease (OR 0.65, 95%CI[0.54, 0.78]), stable high (OR 0.53, 95%CI[0.43, 0.65]), and increase (OR 0.73, 95%CI[0.60, 0.88]) are less likely to maintain stable pre‐frail/frail (model 3; Figure 4 and Table S9).

### Vitamin D and frailty state transitions

Table 4 shows the direction and strength of relationships between an increasing degree of vitamin D and the probabilities of frailty status transitions. The results indicate that a higher degree of vitamin D (estimated in 1 nmol/L) is associated with a lower risk of transitioning from robust to prefrail (HR 0.997, 95% CI [0.995, 0.999]) and from prefrail to frail (HR 0.992, 95% CI [0.988, 0.995]). Additionally, a higher degree of vitamin D is associated with a higher risk of returning to robust status in individuals who were classified as prefrail (HR 1.004, 95% CI [1.001, 1.007]), and a higher risk of returning to prefrail status in those who were classified as frail (HR 1.007, 95% CI [1.002, 1.012]).

**Table 4:** Vitamin D and FI transition probabilities

Transition	HR (95% CI)
Robust to prefrail	0.997 (0.995, 0.999)
Robust to frail	1.101 (0.999, 1.213)
Prefrail to robust	1.004 (1.001, 1.007)
Prefrail to frail	0.992 (0.988, 0.995)
Frail to robust	0.952 (0.139, 6.540)
Frail to prefrail	1.007 (1.002, 1.012)

### Subgroup analyses

The association between vitamin D and frailty remained consistent in subgroup analyses (Tables S10 and S11). Among participants with equal levels of vitamin D, women had a significantly higher probability of being prefrail or frail compared to men, while participants aged ≥60 had a higher probability of frailty (Figures S2 and S3). The restricted cubic spline of the association between vitamin D and frailty status for men and women were shown in Figure S4.

### Sensitivity analyses

The association between baseline vitamin D and incident Frailty events remained consistent in the following analyses: when using IPAW analyses and the complete dataset (Table S12–16).

## Discussion

This study utilized a large prospective database to analyse the association between baseline vitamin D and dynamic changes in vitamin D with frailty status. The key findings of the study are as follows:

(1) Vitamin D insufficiency and deficiency are prevalent in the elderly, affecting 88.7% with insufficient and 13.3% with deficient vitamin D. (2) Pre‐frailty is common in the population, with 51.3–56.8% of individuals experiencing pre‐frail status. (3) Low serum vitamin D levels were associated with a higher likelihood of frailty. There was a non‐linear relationship between baseline vitamin D and frailty and pre‐frailty. The risk was higher when the cutoff points were at 43.618 and 30.729 nmol/L. (4) Vitamin D was associated with weight loss, exhaustion, low physical activity, slow gait speed, and low grip strength. (5) Dynamic changes in vitamin D were related to changes in frailty status. Frailty transition is a dynamic process, and higher vitamin D levels were associated with a lower risk of developing prefrailty and progressing to frailty. Additionally, higher vitamin D levels were associated with a higher possibility of transitioning from frailty to prefrailty and to robust status.

Vitamin D insufficiency and deficiency have become a global issue, with 88.7% of individuals having insufficient levels and 13.3% having deficient levels in this study. Vitamin D deficiency is highly prevalent among older adults, posing a significant threat to their health and quality of life.^19^

The insufficient and deficient levels of vitamin D in older adults may be caused by several factors. Firstly, age‐related decline in kidney function may affect the metabolism and activation of vitamin D.^20^ Second, older adults often have limited outdoor activities and sunlight exposure, which further restricts their ability to naturally obtain vitamin D. Additionally, there may be a decrease in dietary intake of vitamin D among older adults, which contributes to insufficient levels.^21^

The deficiency and insufficiency of vitamin D in older adults may lead to a range of health issues. Vitamin D insufficiency is closely associated with bone health problems, including an increased risk of osteoporosis and fractures.^15^ Furthermore, vitamin D insufficiency is linked to weakened immune function, cardiovascular diseases, diabetes, decreased muscle function, and cognitive decline.^22^, ^23^ Vitamin D plays a role in skeletal muscle, directly affecting muscle strength and function. Studies have shown that vitamin D is related to an increased risk of falls, fractures, and poorer physical performance, which directly influence the process of frailty.^24^

This study found an association between vitamin D and factors such as weight loss, fatigue, low physical activity, slow gait speed, and decreased grip strength, further confirming this observation. The association may involve multiple mechanisms. One possible mechanism is muscle function and energy metabolism. Vitamin D plays a crucial role in maintaining muscle function and energy metabolism. It is involved in regulating muscle contraction, protein synthesis, and skeletal muscle function. Vitamin D deficiency may result in decreased muscle strength, reduced muscle mass, and disrupted energy metabolism, which can impact weight control and physical activity capacity.^25^

Vitamin D deficiency may contribute to mechanisms related to weight loss in the following ways: (1) Vitamin D can influence the secretion of insulin and glucocorticoids, thereby regulating blood glucose levels and affecting satiety.^26^ Additionally, vitamin D can regulate insulin sensitivity, enhance glucose utilization, and reduce fat accumulation.^27^ (2) Vitamin D can impact gene expression in adipocytes, promoting fatty acid oxidation and lipolysis while inhibiting fat synthesis and storage.^28^ (3) Vitamin D can influence the composition and function of the gut microbiota, maintaining its balance.^29^ The gut microbiota plays a crucial role in energy metabolism and weight control.

Inflammation and immune function are also related to vitamin D. Vitamin D has anti‐inflammatory and immune‐regulatory effects.^23^ Vitamin D deficiency may lead to decreased immune function and increased inflammation, resulting in fatigue and decreased physical activity capacity. Obesity is closely linked to inflammation, as individuals with obesity often experience chronic low‐grade inflammation.^30^ Vitamin D can help alleviate inflammation and potentially contribute to weight reduction..^23^

Bone health is another important aspect influenced by vitamin D. Vitamin D deficiency may contribute to osteoporosis and skeletal muscle function decline, which can affect gait speed and grip strength, among other muscle functions.^31^ The nervous system also plays a crucial role in the association between vitamin D and muscle function. Vitamin D is involved in neurodevelopment and the regulation of neural functions, including nerve conduction, neuromuscular coordination, and motor control.^32^ Vitamin D deficiency may affect the functioning of the nervous system, thereby influencing gait speed and grip strength.^33^ These mechanisms may intertwine and interact with each other. Further research is needed to explore the specific mechanisms underlying these associations.

This study indicates that 51.3–56.8% of individuals experience prefrail, and 4.7–8% experience frail. Frailty refers to the loss of physical and mental states, leading to a decrease in the individual's demands for physical activity, cognitive abilities, and emotional states. Frailty can be temporary or persistent. Research suggests that individuals in the pre‐frailty stage are more likely to develop frailty compared to non‐frail individuals, and those in the early stages of frailty are more sensitive to intervention measures. Therefore, it is important to focus on screening for pre‐frailty in older patients and actively provide interventions.

This study demonstrates an association between vitamin D and frailty status, showing a non‐linear relationship. The study found that the risk of frail and pre‐frailty increased when vitamin D levels were below 30.729 and 43.618 nmol/L. Moreover, using a Markov polymorphic model, this study analysed the association between vitamin D and changes in frailty status. The analysis found that for every increase in vitamin D unit, the risk of transitioning from robust to pre‐frailty or frailty decreased. It also found that the likelihood of transitioning from frailty to pre‐frailty increased.

To the best of our knowledge, this study is the first to investigate the association between dynamic changes in vitamin D and changes in frailty status. The analysis found that higher levels of vitamin D were associated with a lower likelihood of transitioning from robust to pre‐frailty/frailty status. Stable high vitamin D levels were also less likely to transition from pre‐frailty to frailty or from frailty to robust/pre‐frailty. Moreover, a decrease in vitamin D levels was less likely to result in a transition from pre‐frailty to robust status, while an increase in vitamin D levels was less likely to result in a transition from pre‐frailty to frailty. Overall, stable high and increasing vitamin D levels were associated with a reduced likelihood of maintaining pre‐frailty/frailty status.

This study offers preliminary evidence of the association between changes in vitamin D and changes in frailty status, providing a basis for a better understanding of the relationship between vitamin D and frailty. It helps uncover the mechanisms by which vitamin D influences frailty and offers a new perspective for the prevention and management of frailty. The findings suggest that changes in vitamin D may be related to changes in frailty status, emphasizing the importance of considering vitamin D as a potential influencing factor in the development and transition of frailty. The results also provide some clues for frailty interventions.

Subgroup analysis revealed that among the study population, females and individuals over the age of 60 had a higher risk of developing frailty at the same levels of vitamin D compared with their counterparts. However, overall, both males and females, regardless of age (>60 or <60), showed an association between vitamin D levels and frailty. This can be attributed to factors such as an increased risk of osteoporosis, hormonal changes during menopause, malnutrition including vitamin D deficiency, reduced outdoor activities, and the presence of chronic diseases and medication use.^15^, ^34^, ^35^ Understanding these factors and their impact on frailty and vitamin D levels is crucial for developing effective interventions and preventive measures for frailty in the elderly, particularly among women.

This study has several strengths, including a large sample size of 444 382 participants from the UK Biobank, which enhances the representativeness and reliability of the findings. Additionally, the study utilized long‐term follow‐up data, allowing for a better understanding of the relationship between vitamin D and frailty, as well as their dynamic changes over time. Furthermore, the study employed multiple assessment methods for frail, providing a more comprehensive and accurate understanding of the relationship.

However, some limitations should be considered. The data source from the UK Biobank may introduce selection bias, as only those who agreed to participate were included. Therefore, the findings may not be universally applicable. Additionally, self‐reported data, such as smoking and alcohol status, sleep duration, etc., may be subject to recall bias or subjective errors, impacting the accuracy of the results. Furthermore, while the study conducted multivariable adjustments, there may still be potential unmeasured confounders, such as genetic factors, other nutritional statuses, etc., which could influence the relationship between vitamin D and frailty.

## Conclusions

This study highlights the importance of vitamin D in the context of frailty. Low levels of vitamin D are associated with an increased risk of frailty, while increasing levels of vitamin D may contribute to improving frailty status. Recognizing the relationship between vitamin D levels and frailty can inform personalized management and early interventions for frail individuals. However, there is still a need for further research to explore the potential effects of vitamin D interventions on frailty and deepen our understanding of the biological connections between vitamin D and frailty. By investigating these connections, we can develop targeted interventions and strategies to mitigate frailty and improve the overall well‐being of older adults.

## Conflict of interest

The authors declared no potential conflicts of interest with respect to the research, authorship, and/or publication of this article.

## Funding

The study was supported by Research Funds of Centre for Leading Medicine and Advanced Technologies of IHM No. 2023IHM01050.

## Supporting information


**Table S1.** Frailty phenotype definition
**Table S2.** Questionnaire items from the baseline UK Biobank assessment used to compose the Frailty Index
**Table S3.** Baseline Characteristics by Frailty index Category
**Table S4.** The demographic characteristics of individuals categorized by serum Vitamin D levels
**Table S5.** Association between baseline categorical Vitamin D and Frailty status
**Table S6**. Association between baseline Vitamin D and individual components of frailty
**Table S7.** Baseline characteristics of the participants according to change in frailty status
**Table S8.** Characteristics of Populations with Varying Serum Vitamin D Levels
**Table S9.** The association between change in Vitamin D and change in frailty status
**Table S10.** Sex stratified Association between baseline Vitamin D and Frailty status
**Table S11.** Age stratified Association between baseline Vitamin D and Frailty status
**Table S12**. Missing data of covariates
**Table S13.** Weighted multivariate‐adjusted OR of Association between baseline Vitamin D and Frailty status
**Table S14.** The association between change in Vitamin D and change in frailty status in IPAW analysis
**Table S15.** Association between baseline Vitamin D and Frailty status in complete data
**Table S16.** The association between change in Vitamin D and change in frailty status in complete data
**Table S17.** Proportion of frailty phenotype in baseline and followup characteristics stratification.
**Figure S1.** The probability of frailty status (stable, pre‐frail, frail) in relation to Vitamin D levels. As Vitamin D concentration increases, the probability of being in the stable category increases, while the probabilities of being pre‐frail and frail decrease.
**Figure S2.** The association between vitamin D and frailty status among men and women.
**Figure S3.** The association between vitamin D and frailty status in participants <60 years and ≥60 years.
**Figure S4.** Restricted cubic spline of the association between vitamin D and frailty status. (A) for prefrail women. (B) for frail women. (C) for prefrail men (D) for frail men
