# Supplementary material for: The association between dynamic changes in vitamin D and frailty alterations: A prospective analysis of UK Biobank participants
Source: J Cachexia Sarcopenia Muscle. 2024 Jun 24;15(5):1722–32. doi: 10.1002/jcsm.13525 (PMC11446684; doi:10.1002/jcsm.13525)
Supplement: Supplementary file 1 — Table S1. Frailty phenotype definition Table S2. Questionnaire items from the baseline UK Biobank assessment used to compose the Frailty Index Table S3. Baseline Characteristics by Frailty index Category Table S4. The demographic characteristics of individuals categorized by serum Vitamin D levels Table S5. Association between baseline categorical Vitamin D and Frailty status Table S6. Association between baseline Vitamin D and individual components of frailty Table S7. Baseline characteristics of the participants according to change in frailty status Table S8. Characteristics of Populations with Varying Serum Vitamin D Levels Table S9. The association between change in Vitamin D and change in frailty status Table S10. Sex stratified Association between baseline Vitamin D and Frailty status Table S11. Age stratified Association between baseline Vitamin D and Frailty status Table S12. Missing data of covariates Table S13. Weighted multivariate‐adjusted OR of Association between baseline Vitamin D and Frailty status Table S14. The association between change in Vitamin D and change in frailty status in IPAW analysis Table S15. Association between baseline Vitamin D and Frailty status in complete data Table S16. The association between change in Vitamin D and change in frailty status in complete data Table S17. Proportion of frailty phenotype in baseline and followup characteristics stratification. Figure S1. The probability of frailty status (stable, pre‐frail, frail) in relation to Vitamin D levels. As Vitamin D concentration increases, the probability of being in the stable category increases, while the probabilities of being pre‐frail and frail decrease. Figure S2. The association between vitamin D and frailty status among men and women. Figure S3. The association between vitamin D and frailty status in participants <60 years and ≥60 years. Figure S4. Restricted cubic spline of the association between vitamin D and frailty status. (A) for prefrail women. (B) for fra [file JCSM-15-1722-s001.docx]

Supplemental Table 1. Frailty phenotype definition

| Individuals  components | Frailty phenotype (used in this manuscript) | UK Biobank  data field |
| --- | --- | --- |
| Weight Loss | Self-reported: “Compared with one year ago, has your weight changed?”  Options:  Yes: weight loss in the previous year.  No: another option. | 2306 |
| Exhaustion | Self-reported: “Over the past two weeks,  how often have you felt tired or had little  energy”  Options:  Yes: more than half time or every day  No: another option | 2080 |
| Low physical activity | Quintiles of sex- age-specific levels of total PA in derived from IPAQ  Options:  Yes: Lowest level of PA  No: low/middle to highest levels of PA | 31, 21022, 22037, 22038, 22039 |
| Slow walking speed | Self-reported: How do you describe your  usual walking pace? (a proxy for gait speed)  Options:  Yes: slow  No: average or brisk pace | 924 |
| Low grip strength | Measured grip strength expressed in kg by sex-and BMI adjusted cut-off points.  Cut-off points:  Men  If BMI ≤24 & grip strength ≤ 29  If BMI 24·1 - 26 & grip strength ≤ 30  If BMI 26·1 - 28 & grip strength ≤ 30  If BMI >28 & grip strength ≤ 32  Women  If BMI ≤23 & grip strength ≤ 17  If BMI 23·1 - 26 & grip strength ≤ 17·3  If BMI 26·1 - 29 & grip strength ≤ 18  If BMI >29 & grip strength ≤ 21 | 31, 21001, 46, 47 |

Supplemental Table 2: Questionnaire items from the baseline UK Biobank assessment used to compose the Frailty Index

| 1. Glaucoma | 18. Diabetes | 35. Any cancer diagnosis |
| --- | --- | --- |
| 2. Cataracts | 19. Coronary heart disease | 36. Multiple cancers diagnosed |
| 3. Hearing difficulty | 20. Angina | 37. Chest pain |
| 4. Migraine | 21. Stroke | 38. Head and/or neck pain |
| 5. Dental problems | 22. High blood pressure | 39. Back pain |
| 6. Self-rated health | 23. Deep-vein thrombosis | 40. Stomach/abdominal pain |
| 7. Fatigue | 24. High cholesterol | 41. Hip pain |
| 8. Sleeplessness/insomnia | 25. Breathing (wheeze) | 42. Knee pain |
| 9. Depressed feelings | 26. Pneumonia | 43. Whole-body pain |
| 10. Self-described nervous  personality | 27. Chronic bronchitis/emphysema | 44. Facial pain |
| 11. Severe anxiety/panic attacks | 28. Asthma | 45. Hypothyroidism |
| 12. Loneliness | 29. Rheumatoid arthriti | 46. Gastric reflux |
| 13. Misery | 30. Osteoarthritis | 47. Hiatus hernia |
| 14. Long-standing illness or disability | 31. Gout | 48. Gall stones |
| 15. Falls | 32. Osteoporosis | 49. Diverticulitis |
| 16. Fractures/broken bones | 33. Hayfever, allergic rhinitis or eczema |  |
| 17. Sciatica | 34. Psoriasis |  |

Supplemental Table 3. Baseline Characteristics by Frailty index Category

| Characteristic | Frailty index, No. (%) | | |
| --- | --- | --- | --- |
|  | Robust | Pre-frail | Frail |
| No. of participants | 196949 (44) | 229444 (51.3) | 21122 (4.7) |
| Age at baseline, years | 56.00 [49.00, 62.00] | 59.00 [51.00, 64.00] | 59.00 [53.00, 64.00] |
| Sex |  |  |  |
| Female | 101217 (51.4) | 126423 (55.1) | 12040 (57.0) |
| Male | 95732 (48.6) | 103021 (44.9) | 9082 (43.0) |
| Townsend deprivation index, median (IQR) | -2.41 [-3.79, -0.08] | -2.02 [-3.57, 0.74] | -0.16 [-2.72, 3.09] |
| Smoking status |  |  |  |
| Never | 116842 (59.5) | 118551 (51.9) | 8702 (41.5) |
| Former | 62782 (32.0) | 84037 (36.8) | 8270 (39.4) |
| Current | 16732 (8.5) | 25900 (11.3) | 4012 (19.1) |
| Alcohol status |  |  |  |
| Never | 7359 (3.7) | 10244 (4.5) | 1652 (7.8) |
| Former | 4420 (2.2) | 9416 (4.1) | 2216 (10.5) |
| Current | 185012 (94.0) | 209481 (91.4) | 17195 (81.6) |
| Ethnicity |  |  |  |
| White | 188284 (95.9) | 218848 (95.7) | 19822 (94.3) |
| Black | 1405 (0.7) | 1504 (0.7) | 142 (0.7) |
| Asian | 3990 (2.0) | 4787 (2.1) | 627 (3.0) |
| Mixed | 1058 (0.5) | 1415 (0.6) | 157 (0.7) |
| Other | 1597 (0.8) | 2075 (0.9) | 270 (1.3) |
| Sleep time |  |  |  |
| Short | 37227 (19.0) | 63590 (27.9) | 8909 (43.2) |
| Normal | 157294 (80.1) | 159402 (70.0) | 10370 (50.3) |
| Long | 1824 (0.9) | 4828 (2.1) | 1327 (6.4) |
| Baseline Vitamin D | 48.30 [33.80, 63.50] | 46.20 [31.90, 61.90] | 39.50 [26.30, 56.10] |

Supplemental Table 4. the demographic characteristics of individuals categorized by serum Vitamin D levels

| Characteristic | ≥75nmol/L | 50-75 nmol/L | 25-50 nmol/L | ≤25 nmol/L |
| --- | --- | --- | --- | --- |
| No. of participants | 50047 (11.3%) | 148926 (33.5%) | 186083 (41.9%) | 59326 (13.3%) |
| Age at baseline, years | 59.00 [51.00, 64.00] | 59.00 [51.00, 64.00] | 57.00 [50.00, 63.00] | 55.00 [48.00, 61.00] |
| Sex |  |  |  |  |
| Female | 26905 (53.8) | 80142 (53.8) | 99597 (53.5) | 31520 (53.1) |
| Male | 23142 (46.2) | 68784 (46.2) | 86486 (46.5) | 27806 (46.9) |
| Townsend deprivation index, median (IQR) | -2.55 [-3.85, -0.49] | -2.48 [-3.81, -0.26] | -2.02 [-3.58, 0.70] | -0.94 [-3.12, 2.18] |
| Smoking status |  |  |  |  |
| Never | 26705 (53.6) | 82277 (55.5) | 102252 (55.2) | 31315 (53.1) |
| Former | 18896 (37.9) | 53867 (36.3) | 63425 (34.2) | 17773 (30.1) |
| Current | 4261 (8.5) | 12228 (8.2) | 19716 (10.6) | 9935 (16.8) |
| Alcohol status |  |  |  |  |
| Never | 1224 (2.4) | 4533 (3.0) | 8146 (4.4) | 4979 (8.4) |
| Former | 1432 (2.9) | 4452 (3.0) | 6771 (3.6) | 3085 (5.2) |
| Current | 47356 (94.7) | 139808 (94.0) | 170919 (92.0) | 51072 (86.4) |
| Ethnicity |  |  |  |  |
| White | 49529 (99.2) | 146081 (98.3) | 176995 (95.5) | 51520 (87.4) |
| Black | 62 (0.1) | 374 (0.3) | 1566 (0.8) | 990 (1.7) |
| Asian | 105 (0.2) | 847 (0.6) | 3706 (2.0) | 4523 (7.7) |
| Mixed | 124 (0.2) | 589 (0.4) | 1251 (0.7) | 647 (1.1) |
| Other | 99 (0.2) | 641 (0.4) | 1858 (1.0) | 1276 (2.2) |
| Sleep time |  |  |  |  |
| Short | 11080 (22.2) | 33902 (22.9) | 46600 (25.2) | 17123 (29.2) |
| Normal | 38026 (76.3) | 112094 (75.6) | 134979 (73.0) | 40066 (68.3) |
| Long | 757 (1.5) | 2256 (1.5) | 3273 (1.8) | 1475 (2.5) |
| Frailty phenotype |  |  |  |  |
| Robust | 20566 (41.1) | 57174 (38.4) | 62585 (33.6) | 16131 (27.2) |
| Prefrail | 26784 (53.5) | 82783 (55.6) | 107628 (57.8) | 35034 (59.1) |
| Frail | 2697 (5.4) | 8969 (6.0) | 15870 (8.5) | 8161 (13.8) |

Supplemental Table 5 association between baseline categorical Vitamin D and Frailty status

| Frailty status | Vitamin D | Model 1,aOR (95% CI) | Model 2,aOR (95% CI) | Model 3,aOR (95% CI) |
| --- | --- | --- | --- | --- |
| Frailty phenotype |  |  |  |  |
| Pre-frail vs Robust | 50−<75 nmol/L vs ≥75 nmol/L | 1.13 [1.10, 1.15] | 1.12 [1.10, 1.15] | 1.05 [1.02, 1.07] |
|  | 25−<50 nmol/L vs ≥75 nmol/L | 1.42 [1.39, 1.45] | 1.37 [1.34, 1.40] | 1.18 [1.16, 1.21] |
|  | < 25 nmol/L vs ≥75 nmol/L | 1.94 [1.88, 1.99] | 1.76 [1.71, 1.81] | 1.41 [1.36, 1.45] |
| Frail vs Robust | 50−<75 nmol/L vs ≥75 nmol/L | 1.20 [1.13, 1.27] | 1.18 [1.11, 1.25] | 1.05 [1.04, 1.06] |
|  | 25−<50 nmol/L vs ≥75 nmol/L | 2.14 [2.03, 2.27] | 1.93 [1.82, 2.04] | 1.38 [1.29, 1.47] |
|  | < 25 nmol/L vs ≥75 nmol/L | 4.77 [4.48, 5.07] | 3.62 [3.39, 3.86] | 2.15 [2.00, 2.31] |
| Frailty index |  |  |  |  |
| Pre-frail vs Robust | 50−<75 nmol/L vs ≥75 nmol/L | 1.04 [1.02, 1.06] | 1.04 [1.01, 1.06] | 1.03 [1.00, 1.05] |
|  | 25−<50 nmol/L vs ≥75 nmol/L | 1.18 [1.15, 1.20] | 1.14 [1.11, 1.16] | 1.11 [1.09, 1.14] |
|  | < 25 nmol/L vs ≥75 nmol/L | 1.41 [1.37, 1.44] | 1.27 [1.24, 1.31] | 1.24 [1.21, 1.28] |
| Frail vs Robust | 50−<75 nmol/L vs ≥75 nmol/L | 1.06 [1.00, 1.12] | 1.04 [0.98, 1.10] | 1.02 [1.00, 1.04] |
|  | 25−<50 nmol/L vs ≥75 nmol/L | 1.67 [1.58, 1.76] | 1.45 [1.37, 1.53] | 1.35 [1.26, 1.44] |
|  | < 25 nmol/L vs ≥75 nmol/L | 3.08 [2.90, 3.26] | 2.13 [2.00, 2.27] | 1.94 [1.80, 2.09] |

Mode 1 adjusted age, sex, ethnicity

Mode 2 adjusted age, sex, ethnicity, smoking status, drinking status, and the Townsend deprivation index

Mode 3 adjusted age, sex, ethnicity, smoking status, drinking status, the Townsend deprivation index, physical activity time, vitamin D supplementation, and sunshine exposure time

Supplemental Table 6 association between baseline Vitamin D and individual components of frailty

| Frailty component | Vitamin D | Model 1,aOR (95% CI) | Model 2,aOR (95% CI) | Model 3,aOR (95% CI) |
| --- | --- | --- | --- | --- |
| Weight loss | 50−<75 nmol/L vs ≥75 nmol/L | 0.87 [0.85, 0.90] | 0.87 [0.85, 0.89] | 0.87 [0.85, 0.90] |
|  | 25−<50 nmol/L vs ≥75 nmol/L | 0.83 [0.81, 0.85] | 0.81 [0.79, 0.83] | 0.82 [0.79, 0.84] |
|  | < 25 nmol/L vs ≥75 nmol/L | 0.87 [0.84, 0.89] | 0.82 [0.79, 0.85] | 0.83 [0.80, 0.87] |
| Exhaustion | 50−<75 nmol/L vs ≥75 nmol/L | 1.14 [1.11, 1.19] | 1.13 [1.09, 1.17] | 1.12 [1.08, 1.17] |
|  | 25−<50 nmol/L vs ≥75 nmol/L | 1.50 [1.45, 1.55] | 1.40 [1.35, 1.45] | 1.35 [1.30, 1.41] |
|  | < 25 nmol/L vs ≥75 nmol/L | 2.07 [2.00, 2.15] | 1.74 [1.67, 1.81] | 1.66 [1.58, 1.73] |
| Low physical activity | 50−<75 nmol/L vs ≥75 nmol/L | 1.17 [1.15, 1.20] | 1.17 [1.14, 1.20] | 1.14 [1.11, 1.17] |
|  | 25−<50 nmol/L vs ≥75 nmol/L | 1.51 [1.48, 1.54] | 1.48 [1.45, 1.51] | 1.40 [1.37, 1.43] |
|  | < 25 nmol/L vs ≥75 nmol/L | 2.06 [2.01, 2.11] | 1.94 [1.90, 2.00] | 1.79 [1.74, 1.84] |
| Slow gait speed | 50−<75 nmol/L vs ≥75 nmol/L | 1.12 [1.07, 1.17] | 1.10 [1.05, 1.15] | 1.03 [0.98, 1.09] |
|  | 25−<50 nmol/L vs ≥75 nmol/L | 1.72 [1.64, 1.79] | 1.55 [1.49, 1.62] | 1.38 [1.30, 1.45] |
|  | < 25 nmol/L vs ≥75 nmol/L | 3.01 [2.88, 3.16] | 2.33 [2.23, 2.45] | 1.91 [1.80, 2.03] |
| Low grip strength | 50−<75 nmol/L vs ≥75 nmol/L | 1.13 [1.10, 1.15] | 1.11 [1.09, 1.14] | 1.10 [1.07, 1.13] |
|  | 25−<50 nmol/L vs ≥75 nmol/L | 1.34 [1.31, 1.38] | 1.28 [1.25, 1.31] | 1.23 [1.20, 1.26] |
|  | < 25 nmol/L vs ≥75 nmol/L | 1.75 [1.70, 1.79] | 1.55 [1.50, 1.59] | 1.45 [1.40, 1.50] |

Mode 1 adjusted age, sex, ethnicity

Mode 2 adjusted age, sex, ethnicity, smoking status, drinking status, and the Townsend deprivation index

Mode 3 adjusted age, sex, ethnicity, smoking status, drinking status, the Townsend deprivation index, physical activity time, vitamin D supplementation, and sunshine exposure time

Supplemental Table 7. Baseline characteristics of the participants according to change in frailty status

| Characteristic | Stable | Robust to pre-frail/frail | Pre-frail to robust | Pre-frail to frail | Frail to robust/pre-frail |
| --- | --- | --- | --- | --- | --- |
| No. of participants | 10462 | 4287 | 1812 | 959 | 423 |
| Age at baseline, years | 58.00 [52.00, 63.00] | 59.00 [52.00, 63.00] | 57.00 [51.00, 62.00] | 59.00 [54.00, 64.00] | 59.00 [53.50, 63.00] |
| Sex |  |  |  |  |  |
| Female | 5205 (49.8) | 2162 (50.4) | 827 (45.6) | 575 (60.0) | 242 (57.2) |
| Male | 5257 (50.2) | 2125 (49.6) | 985 (54.4) | 384 (40.0) | 181 (42.8) |
| Townsend deprivation index, median (IQR) | -2.74 [-3.98, -0.85] | -2.79 [-4.00, -0.89] | -2.95 [-4.11, -1.04] | -2.26 [-3.73, 0.20] | -2.41 [-3.92, 0.08] |
| Smoking status |  |  |  |  |  |
| Never | 6205 (59.5) | 2482 (57.9) | 1067 (59.0) | 553 (58.0) | 236 (55.9) |
| Former | 3583 (34.3) | 1540 (35.9) | 649 (35.9) | 329 (34.5) | 144 (34.1) |
| Current | 646 (6.2) | 263 (6.1) | 93 (5.1) | 72 (7.5) | 42 (10.0) |
| Alcohol status |  |  |  |  |  |
| Never | 310 (3.0) | 102 (2.4) | 43 (2.4) | 60 (6.3) | 19 (4.5) |
| Former | 250 (2.4) | 94 (2.2) | 45 (2.5) | 43 (4.5) | 18 (4.3) |
| Current | 9900 (94.6) | 4090 (95.4) | 1723 (95.1) | 855 (89.2) | 386 (91.3) |
| Ethnicity |  |  |  |  |  |
| White | 10241 (98.1) | 4217 (98.6) | 1778 (98.2) | 922 (96.4) | 412 (97.6) |
| Black | 22 (0.2) | 7 (0.2) | 3 (0.2) | 6 (0.6) | 0 (0.0) |
| Asian | 78 (0.7) | 29 (0.7) | 23 (1.3) | 12 (1.3) | 6 (1.4) |
| Mixed | 45 (0.4) | 11 (0.3) | 1 (0.1) | 8 (0.8) | 1 (0.2) |
| Other | 50 (0.5) | 15 (0.4) | 6 (0.3) | 8 (0.8) | 3 (0.7) |
| Sleep time |  |  |  |  |  |
| Short | 2240 (21.5) | 840 (19.6) | 390 (21.5) | 261 (27.5) | 129 (30.6) |
| Normal | 8050 (77.3) | 3403 (79.5) | 1409 (77.8) | 662 (69.8) | 279 (66.3) |
| Long | 123 (1.2) | 39 (0.9) | 11 (0.6) | 26 (2.7) | 13 (3.1) |
| Baseline Vitamin D | 47.70 [33.50, 62.60] | 50.00 [36.20, 64.10] | 49.00 [34.38, 63.73] | 42.20 [28.50, 56.45] | 45.80 [30.35, 59.95] |

Supplement Table 8. Characteristics of Populations with Varying Serum Vitamin D Levels

| Characteristic | Stable | Increased | Decreased | P |
| --- | --- | --- | --- | --- |
| No. of participants | 12668 | 992 | 947 |  |
| Age at baseline, years | 58.00 [52.00, 63.00] | 60.00 [53.00, 63.00] | 59.00 [52.00, 64.00] | <0.001 |
| Sex |  |  |  | 0.003 |
| Female | 6076 (48.0) | 522 (52.6) | 428 (45.2) |  |
| Male | 6592 (52.0) | 470 (47.4) | 519 (54.8) |  |
| Townsend deprivation index, median (IQR) | -2.72 [-3.96, -0.74] | -2.82 [-3.97, -1.00] | -2.82 [-4.13, -1.31] | 0.004 |
| Smoking status |  |  |  | 0.133 |
| Never | 7479 (59.2) | 597 (60.4) | 540 (57.0) |  |
| Former | 4334 (34.3) | 344 (34.8) | 350 (37.0) |  |
| Current | 825 (6.5) | 48 (4.9) | 57 (6.0) |  |
| Alcohol status |  |  |  | <0.001 |
| Never | 397 (3.1) | 29 (2.9) | 9 (1.0) |  |
| Former | 330 (2.6) | 33 (3.3) | 15 (1.6) |  |
| Current | 11936 (94.3) | 930 (93.8) | 923 (97.5) |  |
| Ethnicity |  |  |  | 0.019 |
| White | 12386 (98.0) | 976 (98.9) | 940 (99.5) |  |
| Black | 29 (0.2) | 2 (0.2) | 0 (0.0) |  |
| Asian | 117 (0.9) | 4 (0.4) | 0 (0.0) |  |
| Mixed | 47 (0.4) | 2 (0.2) | 4 (0.4) |  |
| Other | 64 (0.5) | 3 (0.3) | 1 (0.1) |  |
| Sleep time |  |  |  | 0.25 |
| Short | 2752 (21.8) | 194 (19.6) | 202 (21.4) |  |
| Normal | 9712 (77.0) | 777 (78.6) | 734 (77.8) |  |
| Long | 157 (1.2) | 17 (1.7) | 8 (0.8) |  |
| Frailty phenotype |  |  |  | <0.001 |
| Robust | 5058 (39.9) | 429 (43.2) | 462 (48.8) |  |
| Prefrail | 7004 (55.3) | 518 (52.2) | 449 (47.4) |  |
| Frail | 606 (4.8) | 45 (4.5) | 36 (3.8) |  |

Supplement Table 9. the association between change in Vitamin D and change in frailty status

|  | Robust to pre-frail/frail |  | Pre-frail to robust |  | Pre-frail to frail |  | Frail to robust/pre-frail |  | Stabel prefrail/frail |  |
| --- | --- | --- | --- | --- | --- | --- | --- | --- | --- | --- |
| Model 1 | OR (95%CI) | P | OR (95%CI) | P | OR (95%CI) | P | OR (95%CI) | P | OR (95%CI) | P |
| Decrease | 0.74 [0.61, 0.90] | 0.003 | 0.66 [0.51, 0.86] | 0.002 | 0.86 [0.64, 1.17] | 0.346 | 0.72 [0.46, 1.12] | 0.146 | 0.58 [0.49, 0.70] | <0.001 |
| Stable high | 0.77 [0.63, 0.95] | 0.017 | 0.77 [0.59, 1.01] | 0.058 | 0.48 [0.33, 0.72] | <0.001 | 0.40 [0.21, 0.74] | 0.004 | 0.50 [0.40, 0.61] | <0.001 |
| Increase | 0.81 [0.66, 0.99] | 0.035 | 0.89 [0.69, 1.14] | 0.362 | 0.45 [0.31, 0.66] | <0.001 | 0.57 [0.34, 0.95] | 0.032 | 0.70 [0.59, 0.85] | <0.001 |
| Model 2 | OR (95%CI) | P | OR (95%CI) | P | OR (95%CI) | P | OR (95%CI) | P | OR (95%CI) | P |
| Decrease | 0.76 [0.62, 0.92] | 0.005 | 0.67 [0.52, 0.87] | 0.003 | 0.89 [0.66, 1.21] | 0.482 | 0.75 [0.48, 1.18] | 0.223 | 0.61 [0.51, 0.72] | <0.001 |
| Stable high | 0.79 [0.64, 0.97] | 0.027 | 0.79 [0.60, 1.03] | 0.083 | 0.50 [0.34, 0.75] | 0.001 | 0.42 [0.23, 0.79] | 0.007 | 0.51 [0.41, 0.62] | <0.001 |
| Increase | 0.82 [0.67, 1.00] | 0.047 | 0.90 [0.70, 1.15] | 0.386 | 0.46 [0.31, 0.68] | <0.001 | 0.59 [0.35, 0.99] | 0.043 | 0.72 [0.59, 0.86] | <0.001 |
| Model3 | OR (95%CI) | P | OR (95%CI) | P | OR (95%CI) | P | OR (95%CI) | P | OR (95%CI) | P |
| Decrease | 0.77 [0.63, 0.93] | 0.007 | 0.72 [0.55, 0.93] | 0.012 | 0.92 [0.67, 1.26] | 0.593 | 0.82 [0.51, 1.32] | 0.419 | 0.65 [0.54, 0.78] | <0.001 |
| Stable high | 0.80 [0.65, 0.99] | 0.040 | 0.81 [0.61, 1.06] | 0.129 | 0.50 [0.33, 0.77] | 0.001 | 0.48 [0.26, 0.90] | 0.022 | 0.53 [0.43, 0.65] | <0.001 |
| Increase | 0.80 [0.65, 0.98] | 0.032 | 0.90 [0.70, 1.16] | 0.419 | 0.48 [0.32, 0.73] | <0.001 | 0.61 [0.36, 1.03] | 0.062 | 0.73 [0.60, 0.88] | 0.001 |

Frail phenotype stale robust and Vitamin D stable low as reference

Mode 1 adjusted age, sex, ethnicity

Mode 2 adjusted age, sex, ethnicity, smoking status, drinking status, and the Townsend deprivation index

Mode 3 adjusted age, sex, ethnicity, smoking status, drinking status, the Townsend deprivation index, physical activity time, vitamin D supplementation, and sunshine exposure time

Supplement Table 10. Sex stratified Association between baseline Vitamin D and Frailty status

|  | Robust | Pre-frail | Frail |
| --- | --- | --- | --- |
| Frailty phenotype |  | OR 95%CI | OR 95%CI |
| Men |  |  |  |
| Model 1,aOR (95% CI) | 1 [Reference] | 0.85 [0.84, 0.86] | 0.61 [0.59, 0.62] |
| Model 2,aOR (95% CI) | 1 [Reference] | 0.87 [0.86, 0.88] | 0.68 [0.67, 0.70] |
| Model 3,aOR (95% CI) | 1 [Reference] | 0.88 [0.87, 0.89] | 0.70 [0.69, 0.72] |
| Women |  |  |  |
| Model 1,aOR (95% CI) | 1 [Reference] | 0.86 [0.85, 0.87] | 0.65 [0.64, 0.66] |
| Model 2,aOR (95% CI) | 1 [Reference] | 0.88 [0.87, 0.88] | 0.70 [0.69, 0.71] |
| Model 3,aOR (95% CI) | 1 [Reference] | 0.89 [0.88, 0.90] | 0.72 [0.71, 0.74] |
| Frailty index |  | OR 95%CI | OR 95%CI |
| Men |  |  |  |
| Model 1,aOR (95% CI) | 1 [Reference] | 0.90 [0.89, 0.91] | 0.67 [0.65, 0.69] |
| Model 2,aOR (95% CI) | 1 [Reference] | 0.93 [0.92, 0.94] | 0.78 [0.76, 0.80] |
| Model 3,aOR (95% CI) | 1 [Reference] | 0.93 [0.92, 0.94] | 0.78 [0.76, 0.80] |
| Women |  |  |  |
| Model 1,aOR (95% CI) | 1 [Reference] | 0.91 [0.90, 0.92] | 0.69 [0.68, 0.71] |
| Model 2,aOR (95% CI) | 1 [Reference] | 0.93 [0.92, 0.94] | 0.78 [0.76, 0.79] |
| Model 3,aOR (95% CI) | 1 [Reference] | 0.93 [0.92, 0.94] | 0.78 [0.76, 0.80] |

Mode 1 adjusted age, ethnicity

Mode 2 adjusted age, ethnicity, smoking status, drinking status, and the Townsend deprivation index

Mode 3 adjusted age, ethnicity, smoking status, drinking status, the Townsend deprivation index, physical activity time, vitamin D supplementation, and sunshine exposure time

Supplement Table 11. Age stratified Association between baseline Vitamin D and Frailty status

|  | Robust | Pre-frail | Frail |
| --- | --- | --- | --- |
| Frailty phenotype |  | OR 95%CI | OR 95%CI |
| Age <60 |  |  |  |
| Model 1,aOR (95% CI) | 1 [Reference] | 0.85 \| [0.84, 0.86] | 0.62 \| [0.60, 0.63] |
| Model 2,aOR (95% CI) | 1 [Reference] | 0.87 \| [0.86, 0.87] | 0.69 \| [0.67, 0.70] |
| Model 3,aOR (95% CI) | 1 [Reference] | 0.88 \| [0.87, 0.88] | 0.70 \| [0.68, 0.71] |
| Age ≥60 |  |  |  |
| Model 1,aOR (95% CI) | 1 [Reference] | 0.86 \| [0.85, 0.87] | 0.65 \| [0.64, 0.66] |
| Model 2,aOR (95% CI) | 1 [Reference] | 0.88 \| [0.87, 0.89] | 0.70 \| [0.69, 0.72] |
| Model 3,aOR (95% CI) | 1 [Reference] | 0.90 \| [0.89, 0.91] | 0.74 \| [0.72, 0.75] |
| Frailty index |  | OR 95%CI | OR 95%CI |
| Age <60 |  |  |  |
| Model 1,aOR (95% CI) | 1 [Reference] | 0.90 \| [0.89, 0.91] | 0.67 \| [0.65, 0.68] |
| Model 2,aOR (95% CI) | 1 [Reference] | 0.93 \| [0.92, 0.93] | 0.77 \| [0.75, 0.79] |
| Model 3,aOR (95% CI) | 1 [Reference] | 0.92 \| [0.92, 0.93] | 0.77 \| [0.75, 0.79] |
| Age ≥60 |  |  |  |
| Model 1,aOR (95% CI) | 1 [Reference] | 0.92 \| [0.91, 0.92] | 0.70 \| [0.69, 0.72] |
| Model 2,aOR (95% CI) | 1 [Reference] | 0.94 \| [0.93, 0.95] | 0.79 \| [0.77, 0.80] |
| Model 3,aOR (95% CI) | 1 [Reference] | 0.94 \| [0.93, 0.95] | 0.80 \| [0.78, 0.82] |

Mode 1 adjusted sex, ethnicity

Mode 2 adjusted sex, ethnicity, smoking status, drinking status, and the Townsend deprivation index

Mode 3 adjusted sex, ethnicity, smoking status, drinking status, the Townsend deprivation index, physical activity time, vitamin D supplementation, and sunshine exposure time

Supplemental Table 12 missing data of covariates

|  | N (%) |
| --- | --- |
| Townsend deprivation index | 544 (0.1) |
| Smoking status | 2244 (0.5) |
| Alcohol status | 1092 (0.2) |
| Ethnicity | 2093 (0.4) |
| Sleep time | 458 (0.1) |

Supplement Table 13. Weighted multivariate-adjusted OR of Association between baseline Vitamin D and Frailty status

|  | Robust | Pre-frail | Frail |
| --- | --- | --- | --- |
| Frailty phenotype |  | OR 95%CI | OR 95%CI |
| Model 1,aOR (95% CI) | 1[Reference] | 0.85 [0.85, 0.86] | 0.63 [0.62, 0.64] |
| Model 2,aOR (95% CI) | 1 [Reference] | 0.87 [0.86, 0.88] | 0.69 [0.68, 0.70] |
| Model 3,aOR (95% CI) | 1 [Reference] | 0.88 [0.88, 0.89] | 0.71 [0.70, 0.72] |
| Frailty index |  |  |  |
| Model 1,aOR (95% CI) | 1 [Reference] | 0.91 [0.90, 0.91] | 0.68 [0.67, 0.69] |
| Model 2,aOR (95% CI) | 1 [Reference] | 0.93 [0.92, 0.94] | 0.77 [0.76, 0.79] |
| Model 3,aOR (95% CI) | 1 [Reference] | 0.93 [0.93, 0.94] | 0.78 [0.77, 0.79] |

Mode 1 adjusted age, sex, ethnicity

Mode 2 adjusted age, sex, ethnicity, smoking status, drinking status, and the Townsend deprivation index

Mode 3 adjusted age, sex, ethnicity, smoking status, drinking status, the Townsend deprivation index, physical activity time, vitamin D supplementation, and sunshine exposure time

Supplement Table 14. the association between change in Vitamin D and change in frailty status in IPAW analysis

|  | Robust to pre-frail/frail | Pre-frail to robust | Pre-frail to frail | Frail to robust/pre-frail | Stable prefrail/frail |
| --- | --- | --- | --- | --- | --- |
| Model 1 | OR 95%CI | OR 95%CI | OR 95%CI | OR 95%CI | OR 95%CI |
| Decrease | 0.74 [0.61, 0.90] | 0.66 [0.51, 0.86] | 0.88 [0.65, 1.19] | 0.71 [0.45, 1.11] | 0.58 [0.49, 0.69] |
| Stable high | 0.77 [0.62, 0.95] | 0.77 [0.58, 1.00] | 0.48 [0.32, 0.71] | 0.39 [0.21, 0.73] | 0.49 [0.40, 0.60] |
| Increase | 0.81 [0.66, 0.98] | 0.89 [0.69, 1.14] | 0.45 [0.31, 0.65] | 0.57 [0.34, 0.95] | 0.71 [0.59, 0.85] |
| Model 2 |  |  |  |  |  |
| Decrease | 0.75 [0.62, 0.91] | 0.67 [0.52, 0.86] | 0.89 [0.66, 1.21] | 0.74 [0.48, 1.17] | 0.60 [0.50, 0.71] |
| Stable high | 0.78 [0.63, 0.96] | 0.78 [0.59, 1.02] | 0.51 [0.34, 0.76] | 0.42 [0.22, 0.78] | 0.51 [0.41, 0.62] |
| Increase | 0.81 [0.67, 0.99] | 0.90 [0.70, 1.15] | 0.48 [0.33, 0.70] | 0.58 [0.35, 0.97] | 0.72 [0.60, 0.87] |
| Model 3 |  |  |  |  |  |
| Decrease | 0.76 [0.63, 0.93] | 0.71 [0.55, 0.92] | 0.91 [0.66, 1.24] | 0.81 [0.51, 1.30] | 0.64 [0.54, 0.77] |
| Stable high | 0.79 [0.64, 0.98] | 0.80 [0.61, 1.05] | 0.51 [0.34, 0.78] | 0.47 [0.25, 0.88] | 0.53 [0.43, 0.66] |
| Increase | 0.80 [0.65, 0.98] | 0.90 [0.70, 1.16] | 0.49 [0.33, 0.73] | 0.60 [0.35, 1.01] | 0.74 [0.61, 0.89] |

Frail phenotype stale robust and Vitamin D stable low as reference

Mode 1 adjusted age, sex, ethnicity

Mode 2 adjusted age, sex, ethnicity, smoking status, drinking status, and the Townsend deprivation index

Mode 3 adjusted age, sex, ethnicity, smoking status, drinking status, the Townsend deprivation index, physical activity time, vitamin D supplementation, and sunshine exposure time

Supplement Table 15. Association between baseline Vitamin D and Frailty status in complete data

|  | Robust | Pre-frail | Frail |
| --- | --- | --- | --- |
| Frailty phenotype |  | OR 95%CI | OR 95%CI |
| Model 1,aOR (95% CI) | 1 [Reference] | 0.79 [0.77, 0.82] | 0.64 [0.57, 0.72] |
| Model 2,aOR (95% CI) | 1 [Reference] | 0.80 [0.77, 0.83] | 0.67 [0.60, 0.75] |
| Model 3,aOR (95% CI) | 1 [Reference] | 0.82 [0.79, 0.85] | 0.71 [0.64, 0.80] |
| Frailty index |  |  |  |
| Model 1,aOR (95% CI) | 1 [Reference] | 0.92 [0.89, 0.95] | 0.79 [0.70, 0.89] |
| Model 2,aOR (95% CI) | 1 [Reference] | 0.93 [0.90, 0.97] | 0.84 [0.74, 0.95] |
| Model 3,aOR (95% CI) | 1 [Reference] | 0.93 [0.90, 0.97] | 0.83 [0.73, 0.93] |

Mode 1 adjusted age, sex, ethnicity

Mode 2 adjusted age, sex, ethnicity, smoking status, drinking status, and the Townsend deprivation index

Mode 3 adjusted age, sex, ethnicity, smoking status, drinking status, the Townsend deprivation index, physical activity time, vitamin D supplementation, and sunshine exposure time

Supplement Table 16. the association between change in Vitamin D and change in frailty status in complete data

|  | Robust to pre-frail/frail | Pre-frail to robust | Pre-frail to frail | Frail to robust/pre-frail | Stable prefrail/frail |
| --- | --- | --- | --- | --- | --- |
| Model 1 | OR 95%CI | OR 95%CI | OR 95%CI | OR 95%CI | OR 95%CI |
| Decrease | 0.75 [0.62, 0.91] | 0.67 [0.52, 0.87] | 0.88 [0.65, 1.20] | 0.72 [0.46, 1.13] | 0.59 [0.49, 0.71] |
| Stable high | 0.78 [0.63, 0.96] | 0.78 [0.59, 1.02] | 0.48 [0.32, 0.71] | 0.40 [0.22, 0.75] | 0.50 [0.40, 0.61] |
| Increase | 0.81 [0.66, 0.99] | 0.89 [0.69, 1.14] | 0.43 [0.29, 0.64] | 0.58 [0.35, 0.96] | 0.71 [0.59, 0.85] |
| Model 2 |  |  |  |  |  |
| Decrease | 0.76 [0.62, 0.92] | 0.67 [0.52, 0.87] | 0.89 [0.66, 1.21] | 0.76 [0.48, 1.19] | 0.61 [0.51, 0.72] |
| Stable high | 0.79 [0.64, 0.97] | 0.79 [0.60, 1.03] | 0.50 [0.34, 0.75] | 0.42 [0.23, 0.79] | 0.51 [0.41, 0.62] |
| Increase | 0.82 [0.67, 1.00] | 0.89 [0.70, 1.15] | 0.46 [0.31, 0.68] | 0.59 [0.35, 0.98] | 0.72 [0.59, 0.86] |
| Model 3 |  |  |  |  |  |
| Decrease | 0.77 [0.63, 0.93] | 0.72 [0.55, 0.93] | 0.92 [0.67, 1.26] | 0.82 [0.51, 1.32] | 0.65 [0.54, 0.78] |
| Stable high | 0.80 [0.65, 0.99] | 0.81 [0.61, 1.06] | 0.51 [0.33, 0.77] | 0.48 [0.26, 0.90] | 0.53 [0.43, 0.65] |
| Increase | 0.80 [0.65, 0.98] | 0.90 [0.70, 1.16] | 0.48 [0.32, 0.73] | 0.60 [0.36, 1.03] | 0.73 [0.60, 0.88] |

Frail phenotype stale robust and Vitamin D stable low as reference

Mode 1 adjusted age, sex, ethnicity

Mode 2 adjusted age, sex, ethnicity, smoking status, drinking status, and the Townsend deprivation index

Mode 3 adjusted age, sex, ethnicity, smoking status, drinking status, the Townsend deprivation index, physical activity time, vitamin D supplementation, and sunshine exposure time

Supplement Table 17. Proportion of frailty phenotype in baseline and followup characteristics stratification.

| Characteristic | Frailty phenotype at baseline | | | | Frailty phenotype at follow up | | | |
| --- | --- | --- | --- | --- | --- | --- | --- | --- |
|  | Robust | Pre-frail | Frail | P | Robust | Pre-frail | Frail | P |
| Age |  |  |  | <0.001 |  |  |  | <0.001 |
| <60 | 96089 (38.0) | 139176 (55.0) | 17873 (7.1) |  | 2998 (30.3) | 6129 (61.9) | 782 (7.9) |  |
| ≥60 | 60367 (31.6) | 113053 (59.1) | 17824 (9.3) |  | 1926 (24.0) | 5346 (66.5) | 762 (9.5) |  |
| Sex |  |  |  | <0.001 |  |  |  | <0.001 |
| Female | 77129 (32.4) | 138814 (58.3) | 22221 (9.3) |  | 2076 (23.0) | 6003 (66.6) | 932 (10.3) |  |
| Male | 79327 (38.5) | 113415 (55.0) | 13476 (6.5) |  | 2848 (31.9) | 5472 (61.3) | 612 (6.9) |  |
| Townsend deprivation index |  |  |  | <0.001 |  |  |  | <0.001 |
| Low | 84892 (38.3) | 124330 (56.0) | 12623 (5.7) |  | 3134 (29.2) | 6831 (63.6) | 778 (7.2) |  |
| High | 71409 (32.2) | 127578 (57.5) | 23016 (10.4) |  | 1786 (24.8) | 4639 (64.5) | 765 (10.6) |  |
| Smoking status |  |  |  | <0.001 |  |  |  | <0.001 |
| Never | 88097 (36.3) | 136966 (56.5) | 17486 (7.2) |  | 2977 (28.2) | 6725 (63.8) | 841 (8.0) |  |
| Former | 54024 (35.1) | 87620 (56.9) | 12317 (8.0) |  | 1649 (26.4) | 4019 (64.4) | 577 (9.2) |  |
| Current | 14030 (30.4) | 26507 (57.4) | 5603 (12.1) |  | 292 (26.2) | 704 (63.1) | 120 (10.8) |  |
| Alcohol status |  |  |  | <0.001 |  |  |  | <0.001 |
| Never | 4195 (22.2) | 11347 (60.1) | 3340 (17.7) |  | 107 (20.0) | 346 (64.8) | 81 (15.2) |  |
| Former | 3746 (23.8) | 9006 (57.2) | 2988 (19.0) |  | 94 (20.9) | 272 (60.4) | 84 (18.7) |  |
| Current | 148463 (36.3) | 231474 (56.6) | 29218 (7.1) |  | 4722 (27.9) | 10854 (64.0) | 1378 (8.1) |  |
| Ethnicity |  |  |  | <0.001 |  |  |  | <0.001 |
| White | 151795 (35.8) | 239923 (56.6) | 32407 (7.6) |  | 4846 (27.6) | 11233 (63.9) | 1491 (8.5) |  |
| Black | 664 (22.2) | 1843 (61.6) | 485 (16.2) |  | 9 (23.7) | 21 (55.3) | 8 (21.1) |  |
| Asian | 1845 (20.1) | 5626 (61.3) | 1710 (18.6) |  | 36 (24.3) | 95 (64.2) | 17 (11.5) |  |
| Mixed | 897 (34.4) | 1465 (56.1) | 249 (9.5) |  | 13 (19.7) | 38 (57.6) | 15 (22.7) |  |
| Other | 854 (22.0) | 2382 (61.5) | 638 (16.5) |  | 15 (18.3) | 58 (70.7) | 9 (11.0) |  |
| Sleep time |  |  |  | <0.001 |  |  |  | <0.001 |
| Short | 32575 (30.0) | 63902 (58.8) | 12228 (11.2) |  | 937 (24.3) | 2490 (64.5) | 433 (11.2) |  |
| Normal | 122405 (37.6) | 182271 (56.1) | 20489 (6.3) |  | 3952 (28.6) | 8813 (63.8) | 1038 (7.5) |  |
| Long | 1314 (16.9) | 4327 (55.8) | 2120 (27.3) |  | 30 (14.2) | 130 (61.3) | 52 (24.5) |  |
| Baseline Vitamin D |  |  |  | <0.001 |  |  |  | <0.001 |
| <75 | 135890 (34.5) | 225445 (57.2) | 33000 (8.4) |  | 4255 (26.7) | 10263 (64.3) | 1432 (9.0) |  |
| ≥75 | 20566 (41.1) | 26784 (53.5) | 2697 (5.4) |  | 669 (33.6) | 1212 (60.8) | 112 (5.6) |  |


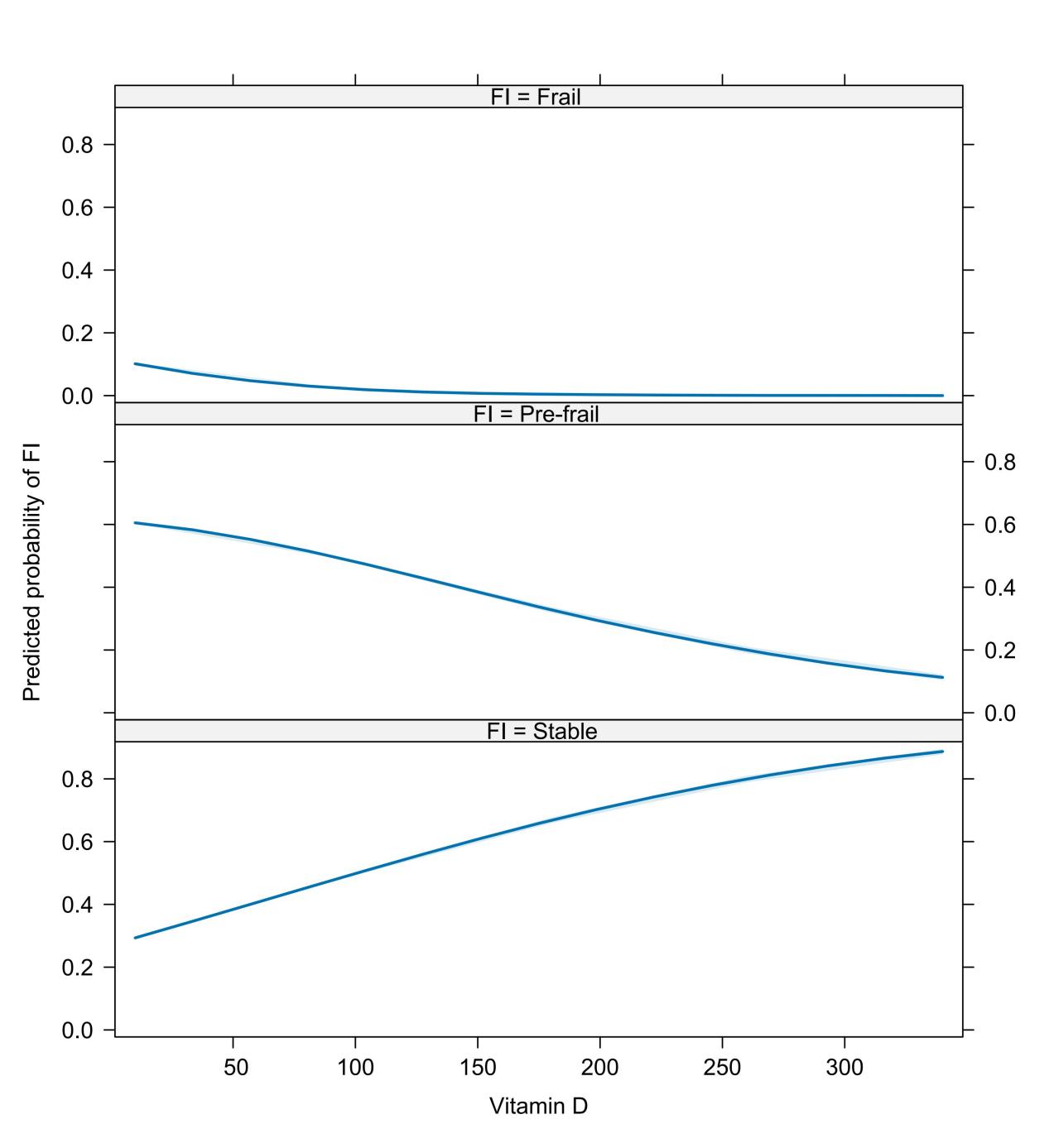


Figure S1. the probability of frailty status (stable, pre-frail, frail) in relation to Vitamin D levels. As Vitamin D concentration increases, the probability of being in the stable category increases, while the probabilities of being pre-frail and frail decrease.


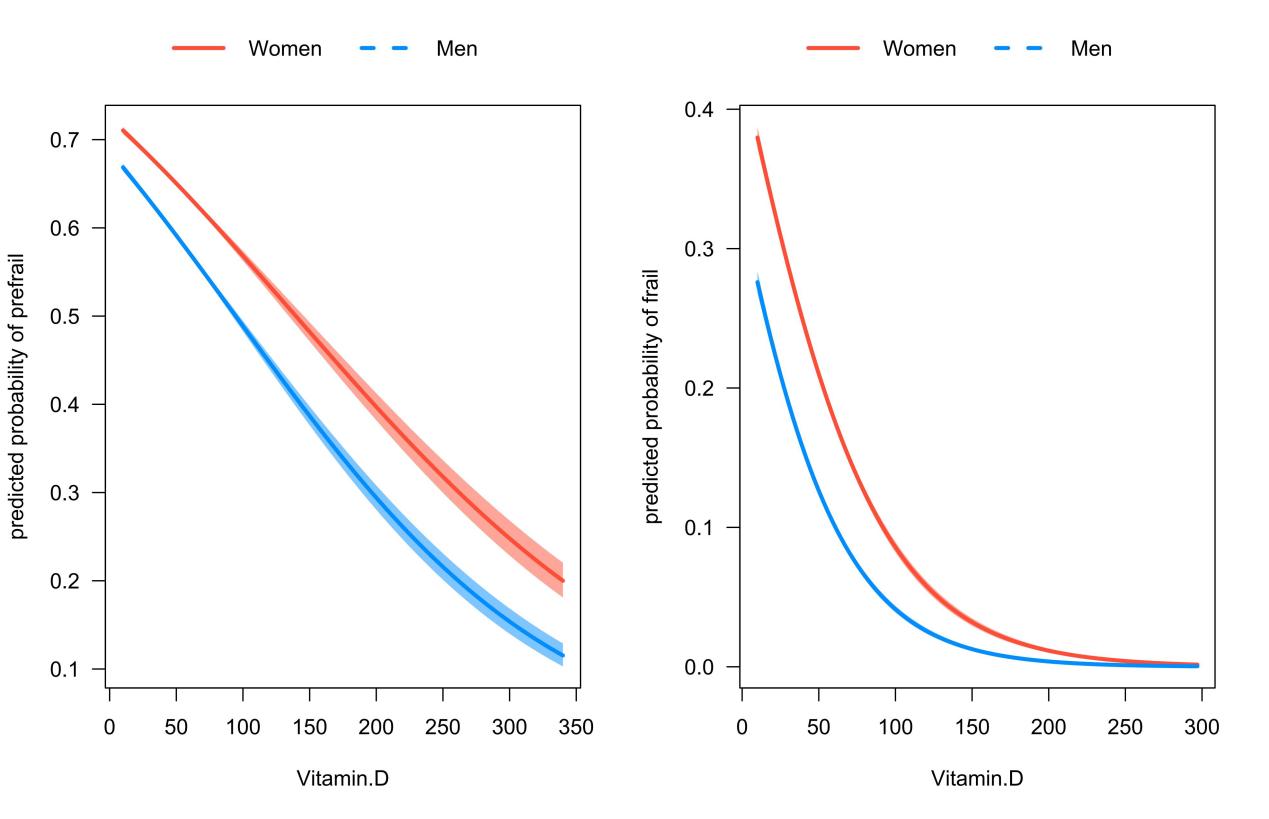


Figure S2. The association between vitamin D and frailty status among men and women.


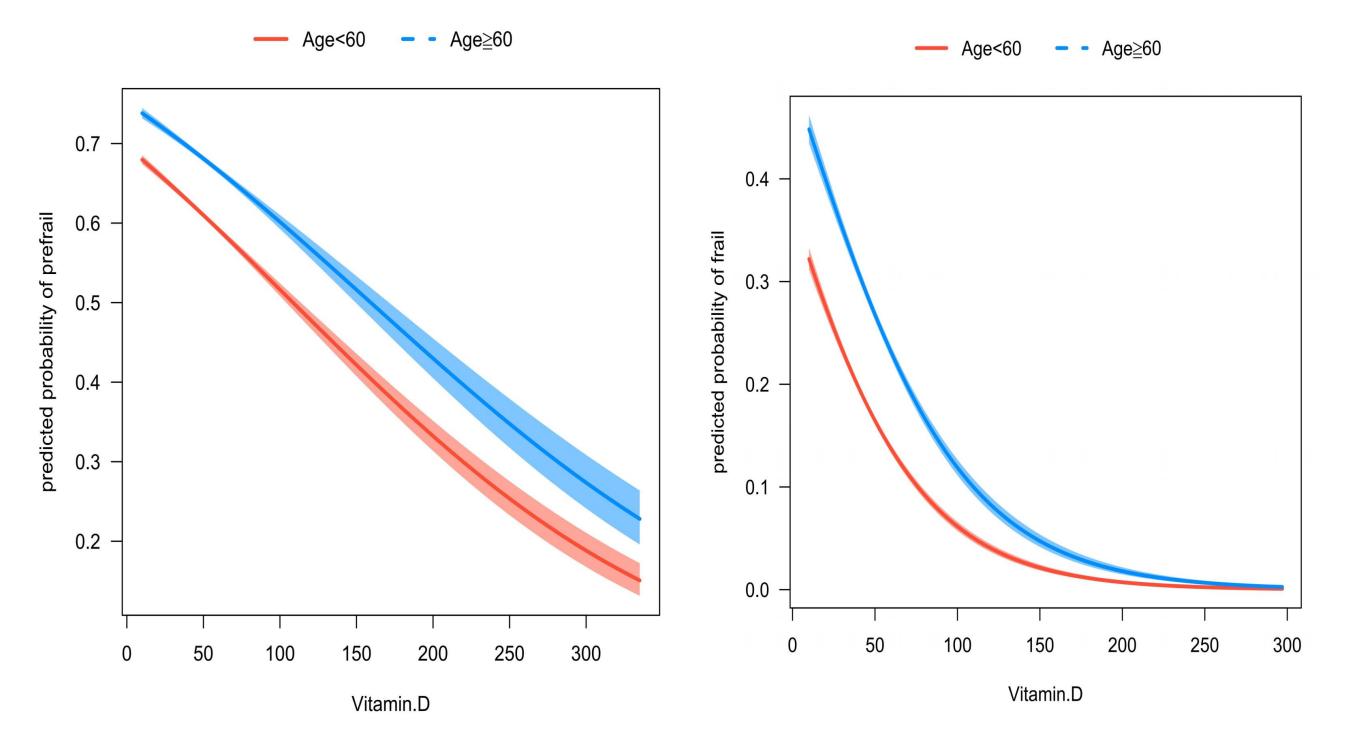


Figure S3. The association between vitamin D and frailty status in participants <60 years and ≥60 years.


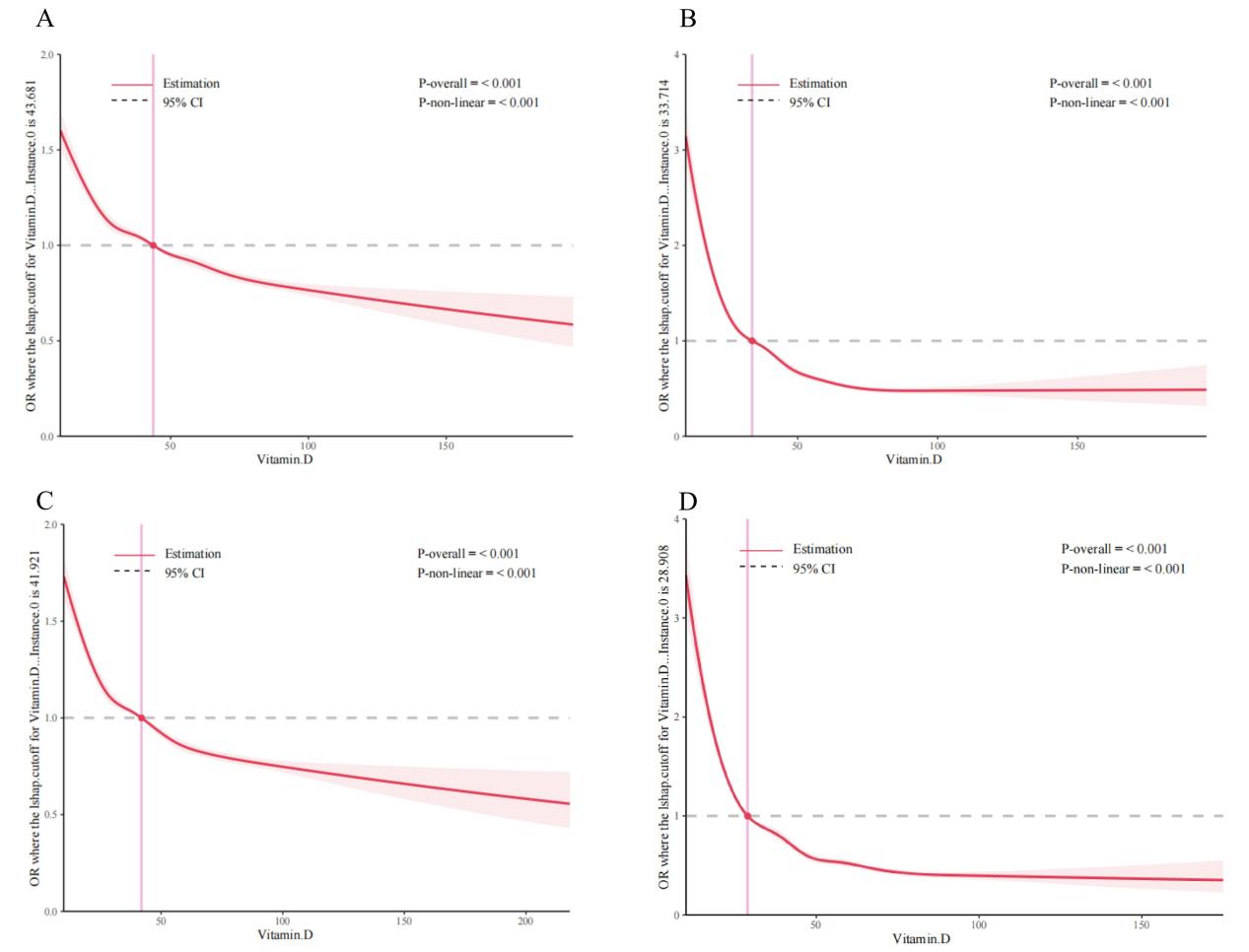


Figure S4. Restricted cubic spline of the association between vitamin D and frailty status. (A) for prefrail women. (B) for frail women. (C) for prefrail men (D) for frail men
